# The efficacy of music therapy for post-stroke depression: A meta-analysis

**DOI:** 10.1097/MD.0000000000044949

**Published:** 2025-10-24

**Authors:** Yan Li, Yanmei Peng, Shiwen Ma

**Affiliations:** a Guilin University of Aerospace Technology, Guilin, Guangxi, China.

**Keywords:** 5-HT, HAMD, meta-analysis, music, post-stroke depression, PSD

## Abstract

**Background:**

Music can stimulate the central nervous system and may exert calming, analgesic, and negative emotion-reducing effects. It has been applied in the treatment of various psychological disorders, including post-stroke depression (PSD). This study systematically assesses the efficacy of music therapy in improving depressive symptoms in patients with PSD.

**Methods:**

A comprehensive search was conducted in 9 databases including Web of Science, PubMed, EMBASE, CNKI, VIP, and Wanfang, covering all publications up to January 7, 2024. Two researchers independently screened articles on music therapy interventions for PSD. Quality assessment and meta-analysis were performed using RevMan 5.3.

**Results:**

A total of 37 randomized controlled trials with 2776 patients were included in the study. Meta-analysis showed that music was effective in improving Hamilton depression scale scores (mean differences [MD] = −4.76, 95% confidence interval [CI]: −6.11 to −3.40, *P* < .00001), Zung Self-Rating Depression Scale scores (MD = −5.25, 95% CI: −6.20 to −4.30, *P* < .00001), Zung Self-Rating Anxiety Scale scores (MD = −7.34, 95% CI: −8.71 to −5.97, *P* < .00001), Barthel index (MD = 13.59, 95% CI: 6.83–20.35, *P* < .00001, activities of daily living scores (MD = 13.09, 95% CI: 4.12–22.05, *P* < .00001), neurological deficit score (standardized mean difference = −1.62, 95% CI: −1.88 to −1.35, *P* < .00001), 5-hydroxytryptamine (MD = 0.86, 95% CI: 0.56–1.16, *P* < .00001) in PSD patients compared to the conventional rehabilitation group.

**Conclusion:**

Music therapy has demonstrated significant clinical efficacy in improving depressive symptoms, daily living skills, the degree of neurological deficits, and serum 5-hydroxytryptamine levels in individuals with PSD.

## 1. Introduction

Stroke is the leading cause of death and disability among adults in China, and with the aging population, the incidence and number of cases are gradually increasing, with an incidence rate of about 10.7%.^[1]^ Post-stroke depression (PSD) is one of the common complications among stroke patients, with an incidence rate of about 30% to 50%,^[2]^ and in recent years, reports have indicated an incidence rate of 25% to 70%.^[3]^ The ability of stroke patients to reintegrate into society is not only related to neurological deficits and physical disabilities following brain damage but also closely linked to the patient’s depressive state and severity. Studies have shown that the severity of disability after a stroke is related to the degree of depression.^[4,5]^ Therefore, effectively treating PSD is an urgent problem in clinical practice.

Currently, the clinical treatment of PSD typically involves the use of serotonin-based antidepressants.Common pharmacological interventions for PSD predominantly include selective serotonin reuptake inhibitors, such as fluoxetine and sertraline. These agents are often considered first-line treatments due to their relatively mild side-effect profiles and favorable cardiovascular tolerability. By enhancing synaptic serotonin levels in the brain, they exert their mood-elevating effects, though adverse reactions such as nausea, gastrointestinal discomfort, headache, insomnia, or sexual dysfunction may occur. Another frequently utilized antidepressant is mirtazapine, which is particularly beneficial for patients experiencing appetite loss or sleep disturbances, with sedation and weight gain being its most commonly observed side effects. Overall, pharmacotherapy should be tailored to the individual’s clinical context, balancing efficacy against potential adverse effects, and ideally integrated with psychological support to enhance functional recovery and emotional well-being.

However, these medications often have adverse effects and can be very expensive. Consequently, non-pharmacological treatments continue to develop and progress in the clinical management of PSD, including acupuncture, music therapy, and exercise therapy. Among these, music therapy is a noninvasive and easily acceptable treatment method, primarily used for the treatment of psychological disorders such as anxiety and depression. Studies suggest that music therapy can regulate neuro-endocrine function, stimulate brain activity, and the melodies can resonate emotionally with patients, thereby alleviating depressive moods in individuals with PSD.^[6]^

Therefore, this study used a meta-analysis method to evaluate the efficacy of independent music therapy for patients with PSD, aiming to provide a basis for clinicians in the treatment of PSD.

## 2. Materials and methods

### 2.1. Inclusion and exclusion criteria

#### 2.1.1. Study type

Randomized controlled trials (RCTs).

#### 2.1.2. Study subjects

Patients diagnosed with PSD), diagnosis must meet the Chinese Classification and Diagnostic Criteria of Mental Disorders-3^[7]^ or the Diagnostic and Statistical Manual of Mental Disorders (DSM-III-R, DSM-IV),^[8]^ with Hamilton Depression Scale (HAMD) scores > 7.

#### 2.1.3. Intervention measures

Experimental group: Combination of music therapy on top of the control group treatment; Control group: Oral administration of antidepressants, psychological counseling, acupuncture, Baduanjin qigong, and conventional treatments.

#### 2.1.4. Outcome measures

Primary outcomes: Depression scores using the HAMD; Self-Rating Depression Scale (SDS); and Self-Rating Anxiety Scale (SAS).

Secondary outcomes: Barthel Index; Activities of Daily Living (ADL); Neurological deficit score; and Serum 5-hydroxytryptamine (5-HT) levels.

#### 2.1.5. Exclusion criteria

The exclusion criteria included patients not diagnosed with PSD; inconsistencies in the conventional treatments used by the experimental and control groups; studies from which data cannot be effectively extracted (lacked necessary effect size estimates, presented incomplete or incompatible outcome measures, or failed to report data in a standardized, analyzable format.); duplicate publications; non-Chinese and non-English literature; registered trials without full texts; and animal studies.

### 2.2. Literature search strategy

A computer search was conducted in PubMed, EMBASE, The Cochrane Library, CNKI, VIP, CBM, and WanFang Data databases to collect RCTs on music therapy for PSD, with the search covering from the inception of each database until January 17, 2024. The search strategy combined both subject headings and free words. Chinese search terms included music, PSD, stroke, depression, etc. English search terms included music, stroke, depression, PSD, etc.

### 2.3. Literature screening and data extraction

Two researchers independently screened literature, extracted data, and cross-checked. Discrepancies were resolved by consulting a third party. Retrieved literature was imported into Endnote X9.3.1 to eliminate duplicates; the first round of screening involved reading titles and abstracts; the second round involved downloading and reading full texts to verify compliance with inclusion criteria, and then extracting data from those that met the criteria. Data extraction included basic information of the studies, such as authors, publication date, title; baseline characteristics of the study subjects; treatment measures; key elements of bias risk assessment; and outcome measures and result data.

### 2.4. Bias risk assessment of included studies

Two researchers independently used the Cochrane Collaboration tool to assess the risk of bias in included studies, covering 6 aspects: generation of random sequence; allocation concealment; blinding; data completeness; selective reporting; and other. The levels of bias risk were categorized as “low risk,” “high risk,” and “unclear.” Additionally, the modified Jadad scale was used to assess the quality of the literature, with scores from 1 to 3 considered low quality and 4 to 7 considered high quality. The results of the bias assessment were cross-checked between the 2 researchers.

### 2.5. Statistical analysis

Statistical analysis was performed using Reviewer Manager 5.4.1 software. For count data included in the outcomes, odds ratios (OR) were used as the effect size metric. If the data were continuous variables and from the same assessment method, mean differences (MD) and 95% confidence intervals (CI) were used for statistics. If outcomes were not from the same assessment method, standardized mean differences (SMD) and 95% confidence intervals (CI) were used. Heterogeneity among study results was quantitatively assessed using the *P*-value and *I*^2^ value. If *P* ≥ .10, there was no heterogeneity among the studies; if *P* < .10, there was heterogeneity. If *I*^2^ < 50%, there was slight heterogeneity among the studies, and a fixed-effects model was used for analysis. If *I*^2^ ≥ 50%, the studies exhibited heterogeneity, and a random-effects model was used for the meta-analysis. The significance level for meta-analysis was set at α = 0.05. Sensitivity analysis was conducted by sequentially excluding individual studies to observe if there were any significant changes in the results after the removal of a single study. Potential publication bias was analyzed using funnel plots.

## 3. Results

The meta-analysis was conducted strictly in accordance with the PRISMA 2020 guidelines.

### 3.1. Literature search process

**Figure 1.**
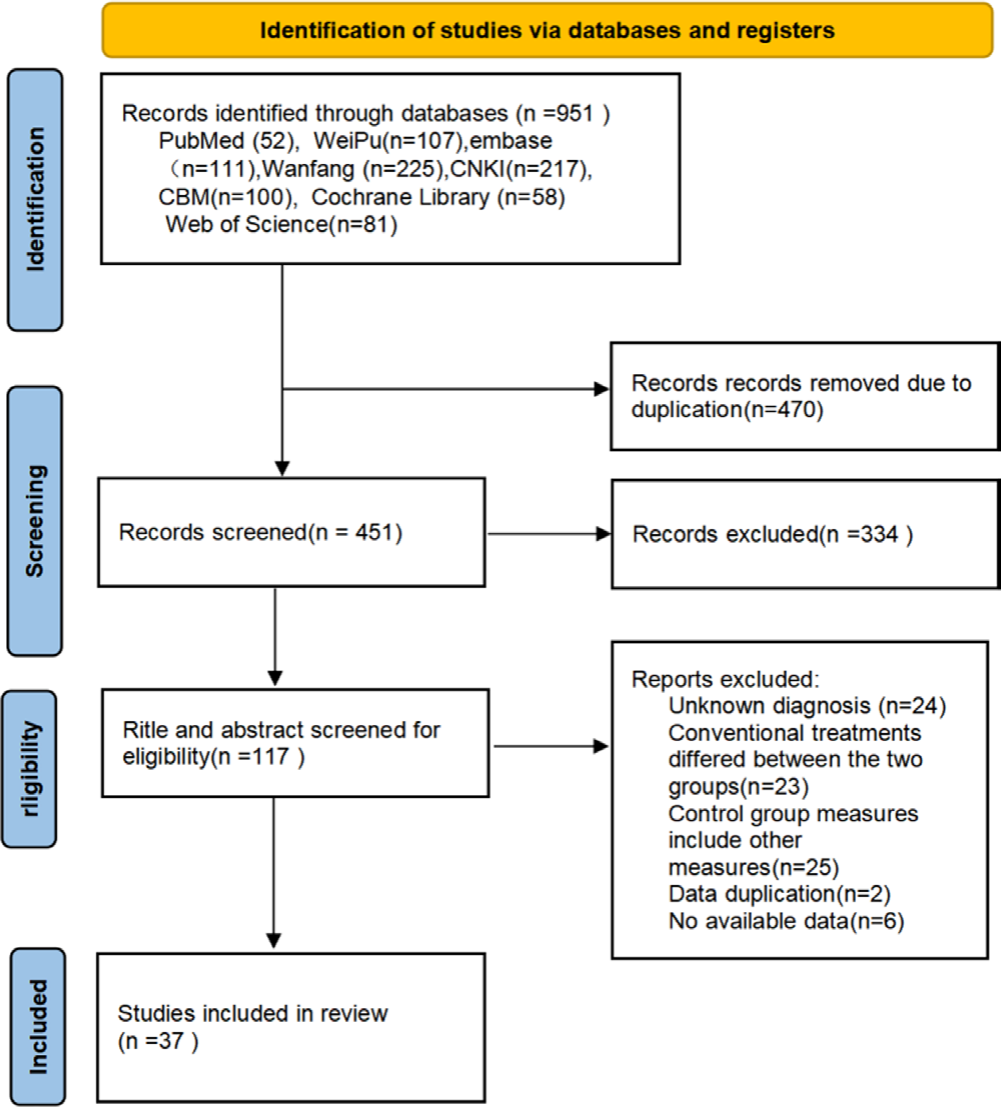
Study selection represented by PRISMA flowchart.

The initial search yielded 951 relevant articles, distributed among the databases as follows: PubMed (n = 52), The Cochrane Library (n = 58), Web of Science (n = 81), Embase (n = 111), WanFang Data (n = 225), CBM (n = 100), CNKI (n = 217), and Weipu VIP (n = 107). After removing duplicates using EndNote, 451 records were included in the initial screening. Upon reading the titles and abstracts, 117 articles were selected for full-text review. Of these, 80 studies did not meet the inclusion criteria and were excluded. Ultimately, 37 RCTs involving 2963 patients were included in the analysis. The literature screening process is illustrated in Figure 1.

### 3.2. Basic information of the included literature

**Figure 2.**
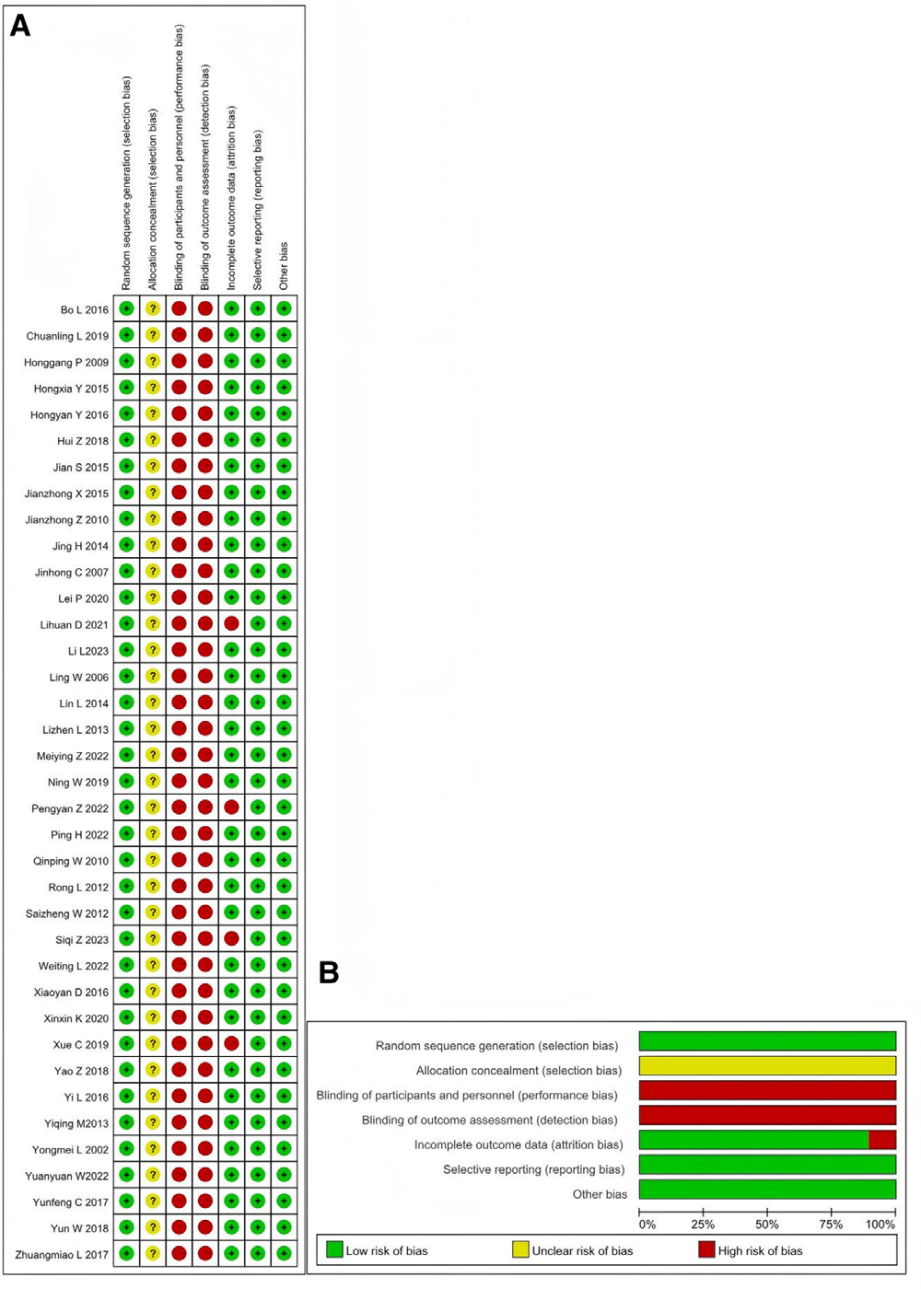
Risk of bias in the included studies.

All 37 included articles^[9–45]^ were RCTs. Among these, 14 articles^[11,13,16,18,22,23,25,31,33,34,37,41,42,44]^ employed random digit table methods, 2 articles^[29,32]^ used the odd-even method, and 1 article^[27]^ used computerized random grouping. The remaining 20 articles did not specify the randomization method. None of the studies described allocation concealment or blinding methods. All reported results were complete. The assessment of bias risk and the percentage representation of each type of bias in the included studies are shown in Figure 2, respectively. According to the modified Jadad scoring criteria, there were 19 high-quality articles and 18 low-quality articles. The basic characteristics of the included studies are presented in Table 1.

**Table 1:** The details of research characteristics.

Study	Sample size(T/C)	Age (T/C)	Type of intervention (T/C)		Intervention frequency	Intervention time	Jadad rating	Outcome measures
Ling Wang^[32]^	30/30	<75/<75	Ⅰ+Ⅲ	Ⅲ	60 min/t,2t/d	2w	2	
Yongmei Li^[21]^	33/30	<75/<75	Ⅰ+Ⅴ	Ⅴ	60 min/t,1t/d	1m	3	
Chuanling Lu^[28]^	50/50	62 ± 7.6/60 ± 8.3	Ⅰ+Ⅱ	Ⅱ	60 min/t,2t/d	56d	4	, ④
Zhuangmiao Li^[22]^	35/35	68.18 ± 52.13/69.22 ± 50.13	Ⅰ+Ⅱ	Ⅱ	60 min/t,1t/d	4w	4	, ⑦
Siqi Zheng^[44]^	47/47	59.74 ± 9.94/58.89 ± 11.50	Ⅰ+Ⅱ	Ⅱ	60 min/t,2t/d	4w	4	, ②
Weiting Liu^[26]^	36/35	59.47 ± 11.96/5860 ± 11.07	Ⅰ+Ⅱ	Ⅱ	30–45 min/t,2t/d,5t/w	4w	3	, ②
Li Liu^[25]^	36/36	60.39 ± 6.46/59.11 ± 5.39	Ⅰ+Ⅱ	Ⅱ	30 min/t,1t/d,5t/w	4w	4	①, ⑥
Lihua Dong^[14]^	35/35	59.15 ± 9.58/57.40 ± 10.16	Ⅰ+Ⅱ	Ⅱ	30–40 min/t,2t/d	2w	3	, ②
Xiaoyan Duan^[15]^	25/25	54.9 ± 9.3/56.3 ± 8.6	Ⅰ+Ⅴ	Ⅴ	30 min/t,1t/d	3w	3	①
Jing Hu^[17]^	30/30	59.43 ± 5.76/59.16 ± 6.68	Ⅰ+Ⅲ	Ⅲ	30 min/t,1t/d	4w	4	②
Yi Lin^[23]^	40/40	66.23 ± 6.74/64.43 ± 6.89	Ⅰ+Ⅲ	Ⅲ	40–60 min/t,1t/d	4w	4	, ④
Lizhen Le^[19]^	75/75	57.6 ± 5.2/57.6 ± 5.2	Ⅰ+Ⅲ	Ⅲ	30 min/t,1t/d,5t/w	4w	4	①
Jinhong Cui^[12]^	29/29	68.5 ± 3.2/68.5 ± 3.2	Ⅰ+Ⅱ+Ⅲ	Ⅱ+Ⅲ	20–30 min/t,2t/d	20d	3	①
Xinxin Ke^[18]^	34/34	59.00 ± 7.31/59.59 ± 6.46	Ⅰ+Ⅲ	Ⅲ	60 min/t,1t/d	1m	4	①
Rong Lu^[27]^	48/50	62~83/61~82	Ⅰ+Ⅲ	Ⅲ	60 min/t,1t/d	40d	4	, ②
Qinping Wang^[34]^	32/32	45~70/45~70	Ⅰ+Ⅲ	Ⅲ	15 min/t,2t/d	4w	4	①
Saizheng Weng^[36]^	30/30	60.1 ± 7.8/59.3 ± 8.5	Ⅰ+Ⅲ	Ⅲ	40 min/t,2t/d	1m	3	①
Jianzhong Zhu^[45]^	40/40	58.7 ± 9.3/59.4 ± 8.6	Ⅰ+Ⅲ	Ⅲ	60 min/t,1t/d	2m	3	①
Jianzhong Xu^[38]^	35/35	49.89 ± 13.99/53.74 ± 14.66	Ⅰ+Ⅲ	Ⅲ	60 min/t,1t/d,6t/w	62d	3	②
Bo Liu^[24]^	30/30	60.5 ± 12.7/61.1 ± 8.19	Ⅰ+Ⅲ	Ⅲ	30 min/t,1t/d	1m	3	①
Jian Shen^[9]^	40/40	68.23 ± 10.46/69.14 ± 9.82	Ⅰ+Ⅲ	Ⅲ	30 min/t,2t/d,5t/w	1m	3	①
Honggang Pei^[30]^	23/22	64.73 ± 3.64/64.73 ± 3.64	Ⅰ+Ⅲ	Ⅲ	60 min/t,2t/d	2w	3	, ④
Ping Han^[16]^	35/35	71.28 ± 8.69/71.34 ± 8.71	Ⅰ+Ⅲ	Ⅲ	60 min/t,1t/d,5t/w	3w	4	①
Hongyan Yang^[39]^	69/68	62.81 ± 6.99/61.91 ± 7.76	Ⅰ+Ⅲ	Ⅲ	30 min/t,1t/d,5t/w	2w	3	①
Lei Pei^[31]^	60/60	67.33 ± 5.94/67.0 ± 5.73	Ⅰ+Ⅳ	Ⅳ	30 min/t,1t/d	8w	4	, ⑤, ⑦
Hongxia Yue^[40]^	45/45	61.6 ± 1.6/61.6 ± 1.6	Ⅰ+Ⅵ	Ⅵ	30 min/t,1t/d	2w	3	, ④, ⑤
Yuanyuan Wu^[37]^	56/56	57.3 ± 6.7/56.7 ± 6.4	Ⅰ+Ⅳ	Ⅳ	30 min,2t/d,5d/w	4w	4	, ④
Yun Wang^[35]^	30/30	66.4 ± 3.15/67.17 ± 3.32	Ⅰ+Ⅵ	Ⅵ	30 min/t,1t/d	4w	3	①
Ning Wang^[33]^	30/30	49.53 ± 7.23/48.56 ± 7.82	Ⅰ+Ⅵ	Ⅵ	30 min/t,1t/d,5t/w	4w	4	, ④
Pengyan Zhang^[41]^	23/22	58.91 ± 5.93/60.09 ± 6.75	Ⅰ+Ⅵ	Ⅵ	30 min/t,1t/d,5t/w	4w	4	①
Yao Zhang^[42]^	21/21	50.02 ± 7.87/49.98 ± 7.60	Ⅰ+Ⅵ	Ⅵ	30 min/t,1t/d,6t/w	6w	4	, ④
Yunfeng Chen^[11]^	36/36	52.21 ± 5.03/51.83 ± 4.87	Ⅰ+Ⅶ	Ⅶ	30 min/t,2t/d	40d	4	①
Meiying Zhai^[13]^	30/30	58.26 ± 22.3/59.38 ± 24.15	Ⅰ+Ⅳ	Ⅳ	30 min/t,2t/d,5t/w	2w	4	, ②
Lin Li^[20]^	38/38	63.2 ± 3.9/62.4 ± 2.3	Ⅰ+Ⅳ	Ⅳ	30 min/t,1/d,8w	8w	3	, ⑤, ⑥
Hui Zhao^[43]^	47/47	67.58 ± 15.81/68.26 ± 15.33	Ⅰ+Ⅳ	Ⅳ	30–40 min/t,1t/d	4w	4	, ⑥
Xue Chen^[10]^	28/27	54.70 ± 3.20/53.40 ± 2.93	Ⅰ+Ⅳ	Ⅳ	30 min/t,1t/d	8w	3	①, ⑤, ⑥
Yiqing Mao^[29]^	60/60	65.4 ± 7.9/68.6 ± 6.4	Ⅰ+Ⅲ	Ⅲ	20–30 min/t,2t/d	2m	2	①

T = experimental group, C = control group,Ⅰ = music therapy, Ⅱ = routine rehabilitation, Ⅲ = conventional treatment, Ⅳ = Deanxit, Ⅴ = psychological counseling, Ⅵ = acupuncture, Ⅶ = baduanjin, ① = HAMD, ② = ADL, ③ = Barthel index, ④ = SAS,⑤ = SDS, ⑥ = neurological deficit score, ⑦ = 5-HT; d = day, w = week, m = month, t = time.

### 3.3. Impact of music therapy on HAMD

**Figure 3.**
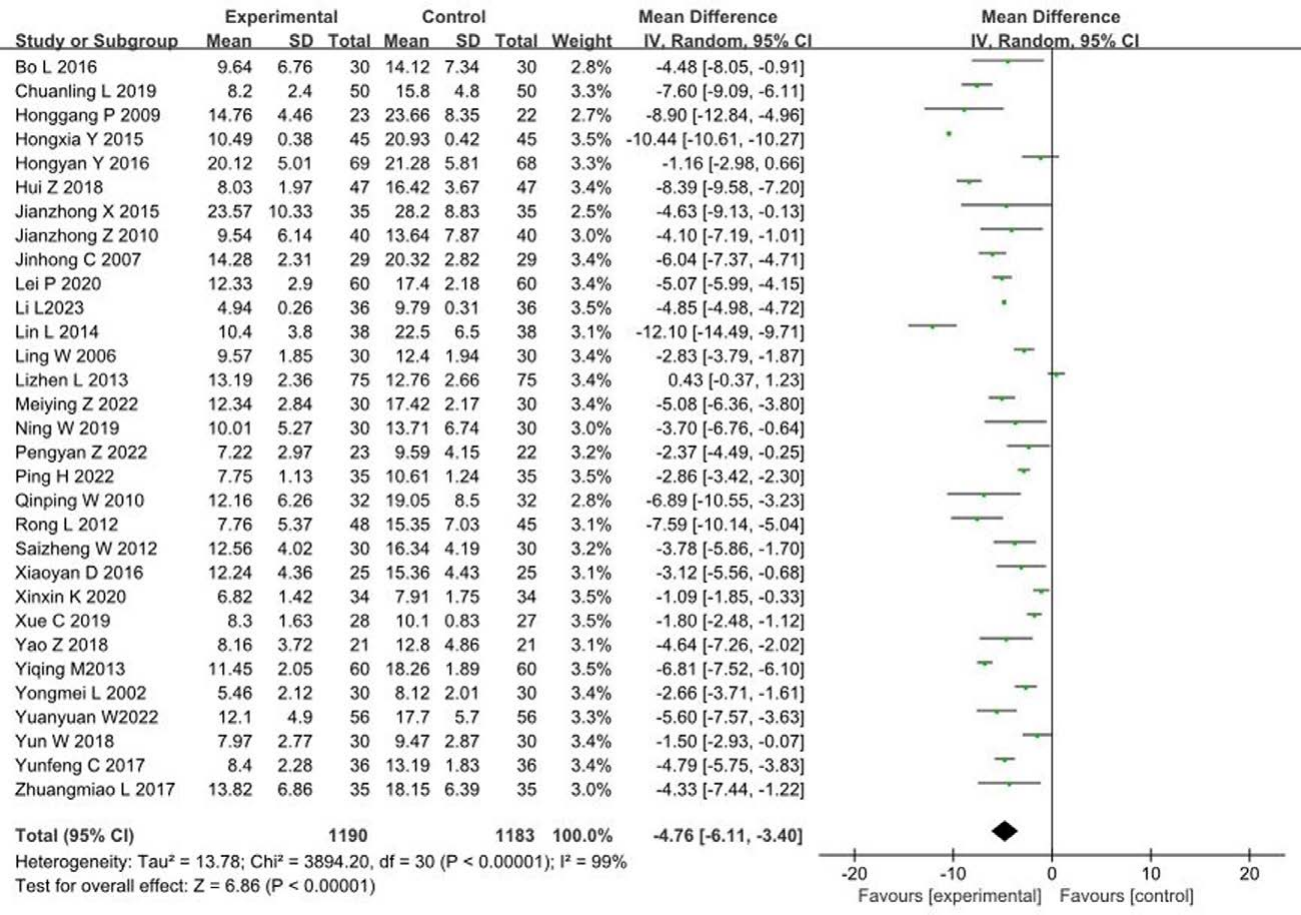
Forest plot of the effects of music therapy on HAMD. CI = confidence interval, HAMD = Hamilton Depression Scale.

A total of 32 articles^[9–13,15,16,18–22,24,25,27–43,45]^ reported on the impact of music therapy on HAMD scores, involving a combined sample of 2373 patients. The heterogeneity test results showed an *I*^2^ > 50% and *P* < .1, indicating the presence of heterogeneity among the studies. Sensitivity analysis was conducted by sequentially excluding individual studies, but no significant sources of heterogeneity were identified. The meta-analysis results, after sequentially excluding and then recombining studies, did not show significant changes compared to the overall results, therefore, a random effects model was used. The meta-analysis results were as follows: (MD = −4.76, 95% CI [−6.11, −3.40], *P* < .00001), indicating that music therapy significantly reduces HAMD scores compared to the control group, as shown in Figure 3.

### 3.4. Impact of music therapy on SDS

**Figure 4.**
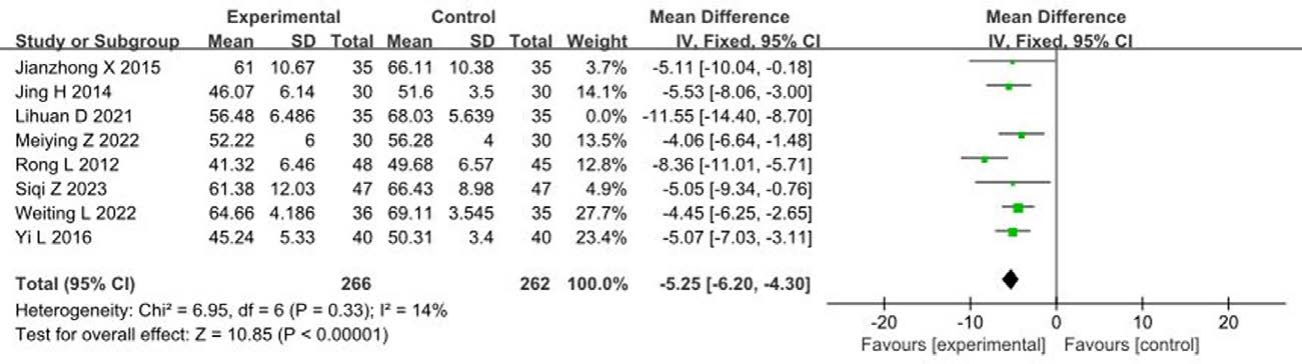
Forest plot of the effects of music therapy on SDS. CI = confidence interval, SDS = Self-Rating Depression Scale.

Eight articles^[13,14,17,23,26,27,38,44]^ reported the effects of music therapy on SDS scores, with a total sample size of 598 patients. The heterogeneity test showed an *I*^2^ > 50% and *P* < .1, indicating heterogeneity among the studies. Sensitivity analysis was conducted by sequentially excluding individual studies; after excluding Lihuan D 2021, the *I*^2^ decreased to 14% and *P* = .33, indicating no significant heterogeneity. Therefore, a fixed-effects model was used. The meta-analysis results were as follows: (MD = −5.25, 95% CI: −6.20 to −4.30, *P* < .00001), indicating that music therapy significantly reduces SDS scores compared to the control group, as illustrated in Figure 4.

### 3.5. Impact of music therapy on SAS

**Figure 5.**
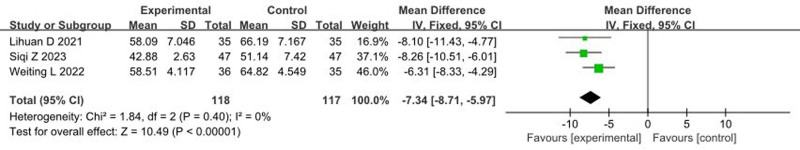
Forest plot of the effects of music therapy on SAS. CI = confidence interval, SAS = Self-Rating Anxiety Scale.

Three articles^[14,26,44]^ reported the effects of music therapy on SAS scores, involving a total of 235 patients. The heterogeneity test showed an *I*^2^ = 0% and *P* = .4, indicating no heterogeneity among the studies. A fixed-effects model was used. The meta-analysis results were as follows: (MD = −7.34, 95% CI: −8.71 to −5.97, *P* < .00001), indicating that music therapy significantly reduces SAS scores compared to the control group, as shown in Figure 5.

### 3.6. Impact of music therapy on Barthel Index

**Figure 6.**
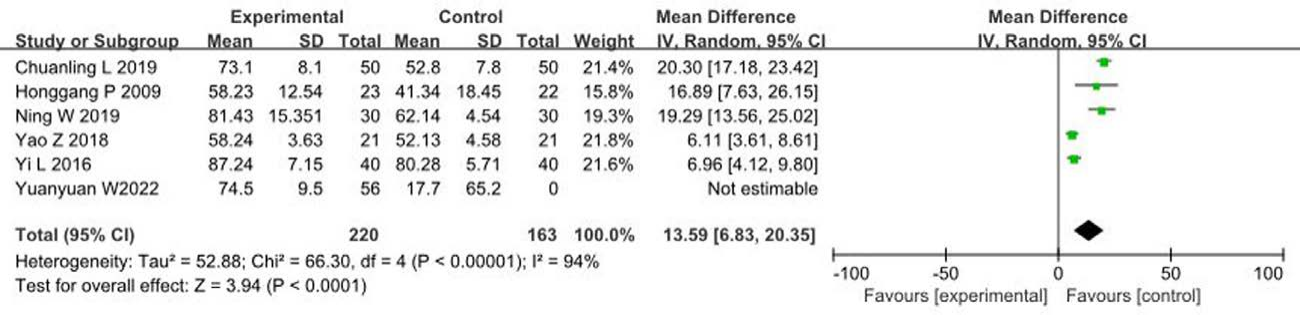
Forest plot of the effects of music therapy on Barthel index. CI = confidence interval.

Six articles^[23,28,30,33,37,42]^ reported the effects of music therapy on the Barthel Index, involving a total of 235 patients. The heterogeneity test showed an *I*^2^ > 50% and *P* < .1, indicating heterogeneity among the studies, thus a random-effects model was used. The meta-analysis results were as follows: MD = 13.59, 95% CI: 6.83 to 20.35, *P* < .00001, indicating that music therapy significantly improves Barthel Index scores compared to the control group, as illustrated in Figure 6.

### 3.7. Impact of music therapy on ADL

**Figure 7.**
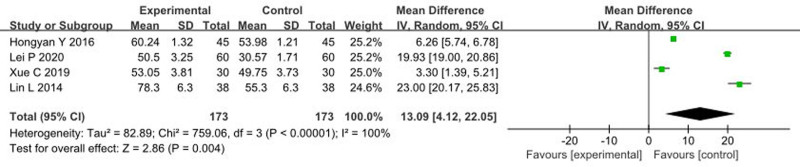
Forest plot of the effects of music therapy on ADL. ADL = activities of daily living, CI = confidence interval.

Four articles^[10,20,31,40]^ reported the effects of music therapy on ADL scores, with a total sample size of 235 patients. The heterogeneity test showed an *I*^2^ > 50% and *P* < .1, indicating heterogeneity among the studies. Sensitivity analysis did not identify significant sources of heterogeneity, and the results of the meta-analysis remained consistent after sequentially excluding and recombining studies. Therefore, a random-effects model was used. The meta-analysis results were as follows: MD = 13.09, 95% CI: 4.12 to 22.05, *P* < .00001, indicating that music therapy significantly enhances ADL scores compared to the control group, as shown in Figure 7.

### 3.8. Impact of music therapy on neurological deficit score

**Figure 8.**
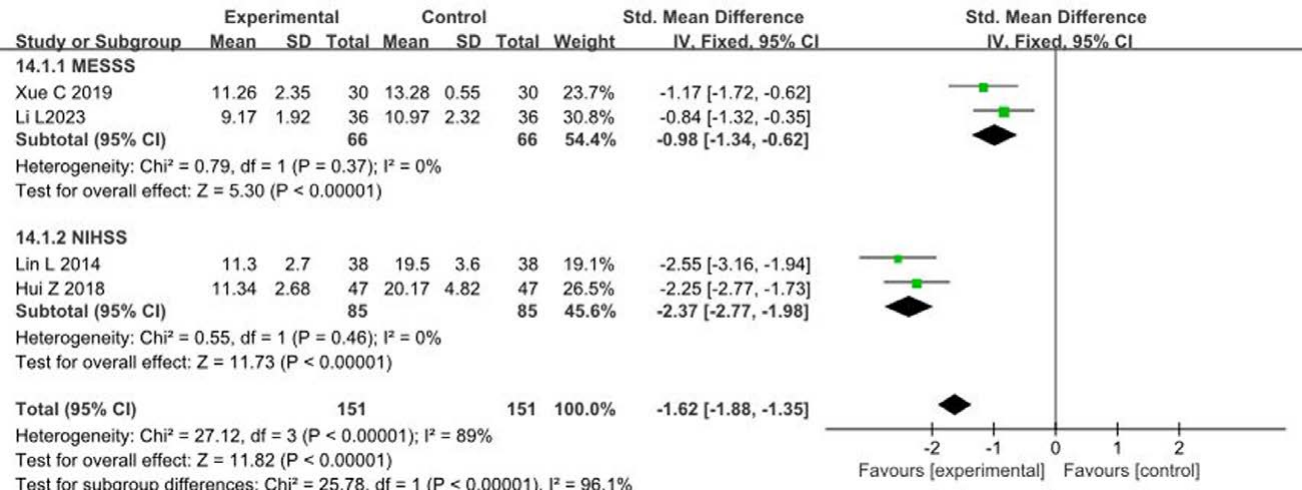
Forest plot of the effects of music therapy on neurological deficit score. CI = confidence interval.

Four articles^[10,20,31,40]^ reported the effects of music therapy on the Neurological Deficit Score, using assessment tools such as MESSS and NIHSS, involving a total of 302 patients. The heterogeneity test showed an *I*^2^ > 50% and *P* < .1, indicating heterogeneity among the studies. Therefore, a random-effects model was used. The meta-analysis results were as follows: standardized mean differences  = −1.62, 95% CI: −1.88, −1.35, *P* < .00001, indicating that music therapy significantly improves Neurological Deficit Scores compared to the control group, as illustrated in Figure 8.

### 3.9. Impact of music therapy on 5-HT levels

**Figure 9.**
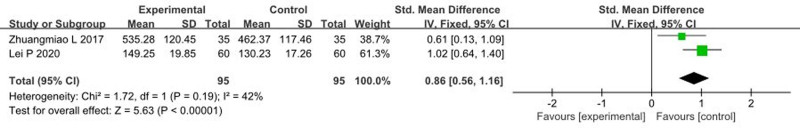
Forest plot of the effects of music therapy on 5-HT. CI = confidence interval, 5-HT = 5-hydroxytryptamine.

Two articles^[22,31]^ reported the effects of music therapy on 5-HT levels, involving a total of 302 patients. The heterogeneity test showed an *I*^2^ = 42% and *P* = .19, indicating no significant heterogeneity among the studies, thus a fixed-effects model was used. The meta-analysis results were as follows: MD = 0.86, 95% CI: 0.56 to 1.16, *P* < .00001, indicating that music therapy significantly increases 5-HT levels compared to the control group, as shown in Figure 9.

### 3.10. Publication bias assessment

**Figure 10.**
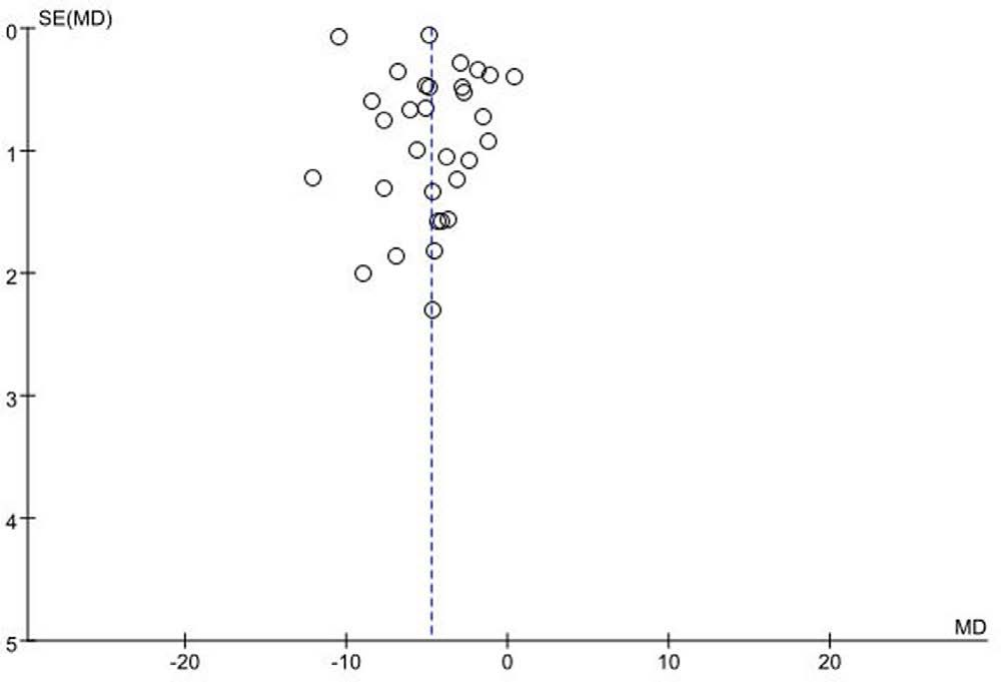
Funnel plot of publication bias analysis based on the HAMD score. HAMD = Hamilton Depression Scale, MD = mean difference.

Due to the limited inclusion of studies on other indicators, we only tested HAMD. The publication bias was assessed through funnel plot analysis based on the improvement of HAMD scores in PSD patients. The results showed a symmetrical distribution on both sides of the funnel plot, indicating no evidence of publication bias, as shown in Figure 10.

## 4. Discussion

PSD is a type of secondary depression that occurs in the months to 1 year after a stroke. It is characterized by persistent low mood, loss of appetite, loss of interest, and decreased energy. In severe cases, it may lead to suicidal thoughts or behaviors and can occur at any stage of the stroke. Music therapy, a noninvasive and economical natural treatment, influences various physiological functions of the human body, thus treating psychosomatic diseases. Modern medicine attributes PSD to an imbalance in monoamine neurotransmitters, particularly a deficiency in 5-HT. Traditional Chinese Medicine (TCM) views stroke as the underlying cause of PSD, which leads to emotional stagnation following the event, described as “depression due to disease (stroke),” involving a complex interplay of pathological factors like wind, phlegm, stasis, and blood leading to qi and blood stagnation. The primary treatment in TCM is to relieve liver qi stagnation.^[46]^

The potential mechanisms by which music therapy alleviates depression in PSD patients include rhythmic music frequencies stimulating the thalamus, amygdala-hippocampus complex, and limbic system, which activates the cerebral cortex, alleviates depression, and improves emotional and physical functions^[47]^; sound waves from music can dilate brain vessels, improve brain blood supply and metabolism, and aid the recovery of damaged brain cells. It also regulates the neuroendocrine system, reduces the expression of catecholamines, and increases endorphin levels, which helps in managing negative emotions and enhancing recovery from depression and anxiety^[48]^; and TCM categorizes natural sounds into 5 tones associated with the 5 elements and organs, which can directly or indirectly affect a person’s emotions and organ functions. Liver qi stagnation is a primary depressive pattern in TCM, with the “spring” tone associated with the liver and wood element, promoting qi movement and emotional relief, thus aiding in sleep and alleviating pain and sorrow.^[49]^

## 5. Conclusion

In summary, based on current evidence, music therapy combined with conventional treatment is superior to conventional treatment alone in improving depression in PSD patients and can somewhat enhance their ability to perform daily activities and aid neurological recovery. This systematic review was limited by the quality and quantity of the included studies, including very few studies with data on SAS, ADL, and 5-HT levels. Future research should employ large-scale randomized controlled trials to optimize testing. Moreover, in-depth discussions considering the differences in patient characteristics and intervention measures should be conducted to validate the conclusions of this study.

## 6. Limitations

Despite a comprehensive analysis and evaluation of all eligible studies, this review has limitations. Firstly, most included studies are of low quality with significant bias risks, potentially affecting the reliability of conclusions. Secondly, only Chinese and no English-language studies were retrieved, limiting the scope of results that could be expanded in the future. Thirdly, factors like the duration of music therapy, session lengths, and types of music used varied across studies, which may have influenced the outcomes. Fourth point, “Standard treatment” remains non-uniform and may encompass a variety of therapeutic combinations, potentially compromising comparability across studies. This limitation has already been acknowledged and addressed in the discussion.

## Author contributions

**Conceptualization:** Yan Li.

**Data curation:** Yanmei Peng.

**Formal analysis:** Yanmei Peng.

**Project administration:** Yan Li.

**Software:** Yanmei Peng.

**Supervision:** Shiwen Ma.

**Validation:** Yan Li.

**Visualization:** Yan Li.

**Writing – original draft:** Yan Li.

**Writing – review & editing:** Shiwen Ma.
